# Rising Midlife Obesity Will Worsen Future Prevalence of Dementia

**DOI:** 10.1371/journal.pone.0099305

**Published:** 2014-09-03

**Authors:** Binod Nepal, Laurie J. Brown, Kaarin J. Anstey

**Affiliations:** 1 National Centre for Social and Economic Modelling, University of Canberra, Canberra, Australian Capital Territory, Australia; 2 Centre for Research on Ageing, Health and Wellbeing, The Australian National University, Canberra, Australian Capital Territory, Australia; University Of São Paulo, Brazil

## Abstract

**Background:**

Midlife body weight status has been found to affect late life dementia outcomes. A cohort projections model was developed to assess the impact of midlife body mass index (BMI) profile on dementia in older Australians.

**Methods:**

A baseline projection using age-sex specific dementia prevalence rates was constructed and the results of scenarios that took account of midlife BMI were compared with those from population ageing only.

**Results:**

This modelling predicts that if the rising trend in midlife obesity and declining trend in midlife normal weight in Australia are to be taken into account in projecting future numbers of Australians with dementia then the number of people aged 65 or more years with dementia, by 2050, would be 14% higher than that expected from demographic ageing only. If midlife obesity prevalence was decreased to 20% and normal weight increased to 40% over the period of 2015–2025, then dementia cases among persons aged 65–69 years would be lower by about 10% in 2050 compared with the “doing nothing to stop current trends in obesity” projection.

**Conclusion:**

The rising tide of obesity in Australian adults will increase the dementia epidemic expected in future years.

## Introduction

Dementia is highly correlated with age. Its prevalence begins to rise from about 1–2 per cent in individuals in their 60s and nearly doubles every five years thereafter [1]. Projections show that, with the ageing of the Australian population, the number of older persons with dementia is expected to increase considerably in the coming decades [2], [3]. These projections are, however, based on the assumptions that age-sex specific prevalence rates remain constant into the future. A question arises as to how the increase in obesity and overweight in Australian adults is likely to influence the future numbers of older persons with dementia. Modelling shows that changes in the risk factor profile of the older population can have substantial upward and downward influences on the number of people with dementia depending on the future trend in the risk factor considered [4]. In the case of obesity, it is midlife rather than late life obesity status that has been shown to matter more with regard to the risk of developing dementia [5]. Many industrialised societies including Australia are experiencing an upward trend in obesity prevalence [6]. This trend indicates that the rising tide of obesity is likely to make the dementia ‘epidemic’ larger than that expected on the basis of demographic ageing only. This study models and examines changes in future projections of Australians aged 65 years and over living with dementia based on past and hypothetical trends in the midlife BMI profile of older persons.

## Method and Data

The modelling began with estimating dementia prevalence by age, sex and midlife BMI status using information on age-sex specific dementia prevalence and prevalence ratios associated with four BMI groups: underweight, normal weight, overweight and obesity. Then the dementia prevalences were applied to the projected population to obtain the number of people with dementia. Population projections were obtained from the Australian Bureau of Statistics [7]. We generated ‘What if’ scenarios by modifying the midlife BMI profile. The age-sex BMI prevalences were obtained by modelling historical time-series data [8]. For the purposes of this study midlife was taken to be 50 years of age.

The modelling steps are described below.

### Step 1: Disaggregating dementia prevalence rates by midlife BMI status

Expressing the prevalence ratio (PR) of dementia in people in category *j* compared to those in the reference category (suffix 1) as
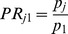
(1)And rearranging, we obtain 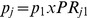
(2)Now, total prevalent cases (*P*) are sum of prevalent cases across *j* categories of a risk factor,
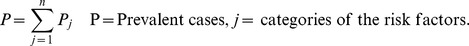


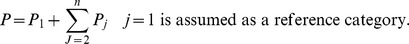


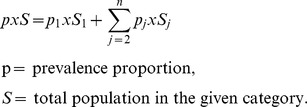
By substitution,
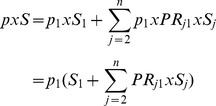
Rearranging, we obtain
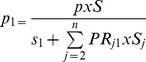
(3)Once prevalence rate for the reference category (p_1_) was estimated, prevalence rates for other categories were estimated using equation (2).

### Step 2: Projecting prevalent cases of dementia

People were classified into four categories of body mass index (BMI) using standard definitions: obesity (BMI≥30), overweight (BMI 25 to <30), normal range (BMI 18.5 to <25), and underweight (BMI<18.5). Age-sex-BMI specific prevalence rates were assumed to remain constant into the future. These rates were applied to population projections disaggregated by age, sex and BMI status.

### Step 3: Comparing ‘ageing only’ and ‘BMI factored’ projections

An ‘ageing-only’ baseline scenario was derived by applying age-sex specific dementia prevalence to the population projections and results of the ‘BMI-factored’ scenario generated by applying age-sex-BMI specific dementia prevalence on the same population projections compared with the baseline scenario. Absolute and relative differences in projected number of people living with dementia based on the ‘ageing-only’ and midlife BMI factored scenarios were calculated. The BMI-factored baseline scenario (Appendix S1) was based on the projection that obesity prevalence at age 50 years would peak at about 46% for males and 39% for females, and normal weight would decline to 13% [8]. This level of obesity is substantially higher than that in in 2010 when about 33% of Australian males and 30% of Australian females aged 50 years were estimated to be obese.

### Step 4: Generating intervention scenarios

Two intervention (“what if”) scenarios were generated by modifying the baseline projection of midlife obesity and normal weight prevalence rates. The results are compared against the ‘ageing only’ and ‘BMI-factored baseline’ scenarios. Thus, four scenarios are examined:

Scenario 1: ageing only: applies age-sex specific dementia prevalence.

Scenario 2: BMI-factored baseline: age-sex-BMI specific dementia prevalence based on baseline midlife trend.

Scenario 3: BMI stabilised: obesity and normal BMI stabilised at 2015 level, an optimistic scenario. The obesity levels were 36% for men and 32.6% for women, and normal weight levels were 22.2% for men and 32.3% for women [8].

Scenario 4: BMI improves: midlife obesity prevalence reduced to 10% and normal BMI increased to 50% over the period of 2015–2025 and maintained at that level thereafter. It was assumed that the BMI profile of the population changes gradually over the intervention period. This scenario illustrates the outcome of reversing the obesity trend back to levels observed in the 1980s [9].

### Data on dementia prevalence

There is no consensus regarding the use of dementia prevalence estimates that best suit the Australian population. A number of different sets of prevalence estimates have been used in the previous studies of dementia in Australia [2], [3], [10]. To capture the high [1] and low [11] ranges of the available prevalence estimates, two sets of age-sex specific dementia prevalence rates have been used in this modelling (Table 1). The age-sex specific prevalence rates were split for the four midlife BMI categories, namely, obese, overweight, normal weight and underweight using the following prevalence ratios: 1.64 for midlife obesity, 1.26 for midlife overweight [5], and 1.2 for midlife underweight [12] versus midlife normal weight.

**Table 1:** Dementia prevalence (%) estimates used in this modelling.

Age	Low prevalence regime [3], [11]		High prevalence regime [1]	
	Male	Female	Male	Female
65–69	1.6	1.0	3.0	4.5
70–74	2.9	3.1	6.2	4.3
75–79	5.6	6.0	10.7	10.6
80–84	11.0	12.6	16.9	16.0
85–89	12.8	20.2	25.1	21.0
90+	22.1	30.8	43.0	41.0

## Results

Table 2 presents results for the four scenarios based on the low and high prevalence regimes for the total population aged 65 years and over. Column 1 shows the numbers of older persons who would be living with dementia according to the ageing-only scenario and as expected these numbers are vastly different depending on the prevalence rates chosen. Columns 2 to 7 compare the outcomes of three BMI-factored scenarios against the ageing-only scenario. If the rising trend in midlife obesity and declining trend in midlife normal weight were to be taken into account, by 2050, the number of people with dementia would be 14% higher than that expected purely from demographic ageing (column 3). Modifying the midlife BMI profile from 2015 would begin to show some impact from 2035. However the impact can barely be noticed when the entire older population aged 65 years and over is considered because a large fraction of older people had already crossed their midlife when the intervention began (columns 4–7).

**Table 2:** Estimated numbers of people aged 65+ years living with dementia under various scenarios and difference to ageing-only scenario.

	Scenario1: Ageing-only	Scenario 2: BMI-factored baseline		Scenario 3: BMI stabilised		Scenario 4: BMI improved	
	Total prevalence cases of dementia	Extra cases to Scenario 1	%increase to Scenario 1	Extra cases to Scenario 1	%increase to Scenario 1	Extra cases to Scenario 1	%increase Scenario 1
	(1)	(2)	(3)	(4)	(5)	(6)	(7)
Low prevalence regime							
2010	202400	0	0.0%	0	0.0%	0	0.0%
2015	236900	4200	1.8%	4200	1.8%	4200	1.8%
2020	277400	10700	3.9%	10700	3.9%	10700	3.9%
2025	328500	19400	5.9%	19400	5.9%	19400	5.9%
2030	393300	31000	7.9%	31000	7.9%	31000	7.9%
2035	461600	44900	9.7%	44500	9.6%	43600	9.4%
2040	527600	59700	11.3%	58700	11.1%	55200	10.5%
2045	582700	74300	12.8%	72000	12.4%	63400	10.9%
2050	631400	88200	14.0%	83200	13.2%	65600	10.4%
High prevalence regime							
2010	318300	0	0.0%	0	0.0%	0	0.0%
2015	377800	6500	1.7%	6500	1.7%	6500	1.7%
2020	444700	16700	3.8%	16700	3.8%	16700	3.8%
2025	526400	30500	5.8%	30500	5.8%	30500	5.8%
2030	623700	48000	7.7%	47900	7.7%	47700	7.6%
2035	725000	68400	9.4%	67700	9.3%	65400	9.0%
2040	825600	90900	11.0%	88800	10.8%	81100	9.8%
2045	906500	112500	12.4%	107900	11.9%	91000	10.0%
2050	984600	133900	13.6%	124800	12.7%	93800	9.5%

Note:

Scenario 2: BMI-factored baseline with prevalence of obesity at age 50 years for males 46% and females 39%, and normal weight 13%.

Scenario 3: Obesity prevalence for males 36% and females 33%, and normal weight for males 22% and females 32% from 2015.

Scenario 4: Starting 2015, obesity decreased to 20% and normal weight increased to 40% by 2025 and maintained at this level thereafter.

**Figure 1:**
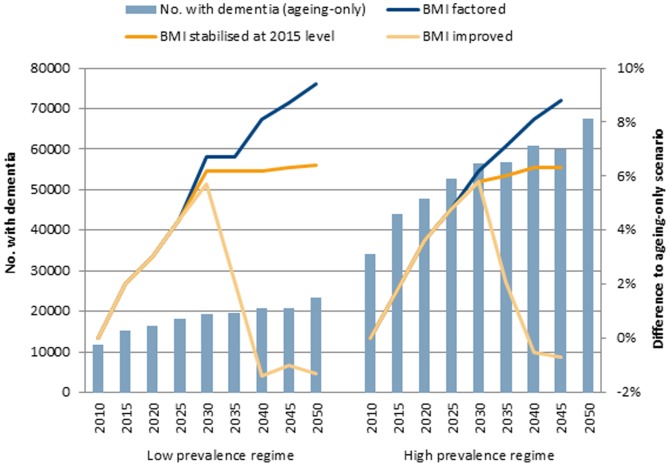
Number of people aged 65–69 years living with dementia and % difference to ageing-only scenario.

Taking the 65–69 year age group as an example, the impact of modifying midlife BMI profile becomes apparent in individuals of this age after 2035 (Figure 1). Compared with Scenario 1 (ageing-only), Scenario 2 (BMI factored baseline) would produce 8–9% more prevalent cases of dementia, and Scenario 3 (BMI stabilised at 2015 level) would produce 6% more cases in people in their late 60s. Scenario 4 (BMI-improved) would reduce dementia prevalence cases close to the level expected from the ageing-only scenario. Compared with Scenario 2 (doing nothing to the prevailing obesity trend), Scenario 4 intervention (reducing obesity prevalence to 20% and raising normal weight to 40%) would lower dementia cases in the 65–69 year age group by 4–5% in 2035 and 9–10% in 2050.

## Discussion and Conclusions

This work illustrates the potential impact of the historical continuing upward trend in midlife obesity on future dementia numbers in Australia. In the current environment characterised by escalating prevalence of obesity and falling prevalence of normal weight, our modelling shows that, in 2050, there could be 14% more people aged 65 years and over living with dementia than that estimated from demographic ageing alone.

Interventions aimed at containing obesity rates in middle-aged adults at the present level are likely to have only a marginal impact on the size of the dementia epidemic expected in the near future. A substantial reduction in the present obesity level in middle-aged Australians will be needed to contain future dementia numbers even to the size expected from demographic ageing alone. Assuming that a decade is needed to implement the BMI interventions and factoring that a decade and a half is needed for these cohorts to age to 65 years, about two decades are elapsed before the impact of the interventions become noticeable. To illustrate this, we looked at the cross-section of people aged 65–69 years. If an intervention is implemented over the period 2015–2025, its impact on dementia becomes noticeable only after 2035. In an illustrative scenario, we estimated that reducing obesity prevalence to 20% and raising normal weight prevalence to 40% by 2025 would reduce dementia cases by 4–5% in 2035 and 9–10% in 2050.

In this modelling, we focused on relative changes rather than absolute number of cases of dementia. By using two sets of prevalence estimates representing a high and a low prevalence regime, we illustrated the uncertainty in the estimates of number of people living with dementia arising from the choice of one or other prevalence rates. It shows that the model is fairly robust to the choice of prevalence rates. The model was developed in Excel 2010 and provides options for replacing the input data such as age-sex specific dementia prevalence rates and prevalence ratios, and for modifying intervention scenarios by altering BMI profile and the intervention period.

This modelling exercise is a modest attempt to incorporate a modifiable lifestyle risk factor into the projection of dementia and therefore advances previous works based on hypothetical assumptions about delaying or eliminating onset of dementia [10], [13]. This work adds a new perspective to a similar work that examined the impact of modifying life style factors on dementia [4] by looking at the impact of changes in midlife body weight profile of birth cohorts.

While this modelling demonstrates that modifying midlife obesity helps contain the future dementia epidemic, the limitations of the study need to be taken into account when interpreting the results. The main limitation is that only BMI was factored in the model. Other lifestyle and biomedical risk factors such as smoking, physical activity, diabetes, cholesterol and blood pressure that have been found to influence the incidence of dementia [14] are not included in the model. Modelling the joint effect of multiple risk factors was not possible due to the lack of historical prevalence data and prevalence ratios for such combinations. Yet even without modelling the joint impact of multiple factors, the findings of this study remain useful as midlife obesity and overweight have been confirmed as influencing the occurrence of dementia in late life independent of a history of some of the other major risk factors such as diabetes and vascular diseases [15].

In conclusion, interventions aimed at reducing obesity and increasing normal BMI in middle-aged people can mitigate the effect of rising obesity on late life dementia. However, the impacts only begin to be noticed in the long term. The lag time needs to be taken into account at the time of planning of such interventions.

## Supporting Information

Appendix S1 Baseline trend of prevalence of midlife obesity, overweight and normal weight.
(TIF)
