# Supplementary figures and images for: Rising Midlife Obesity Will Worsen Future Prevalence of Dementia
Source: PLoS One. 2014 Sep 3;9(9):e99305. doi: 10.1371/journal.pone.0099305 (PMC4153475; doi:10.1371/journal.pone.0099305)

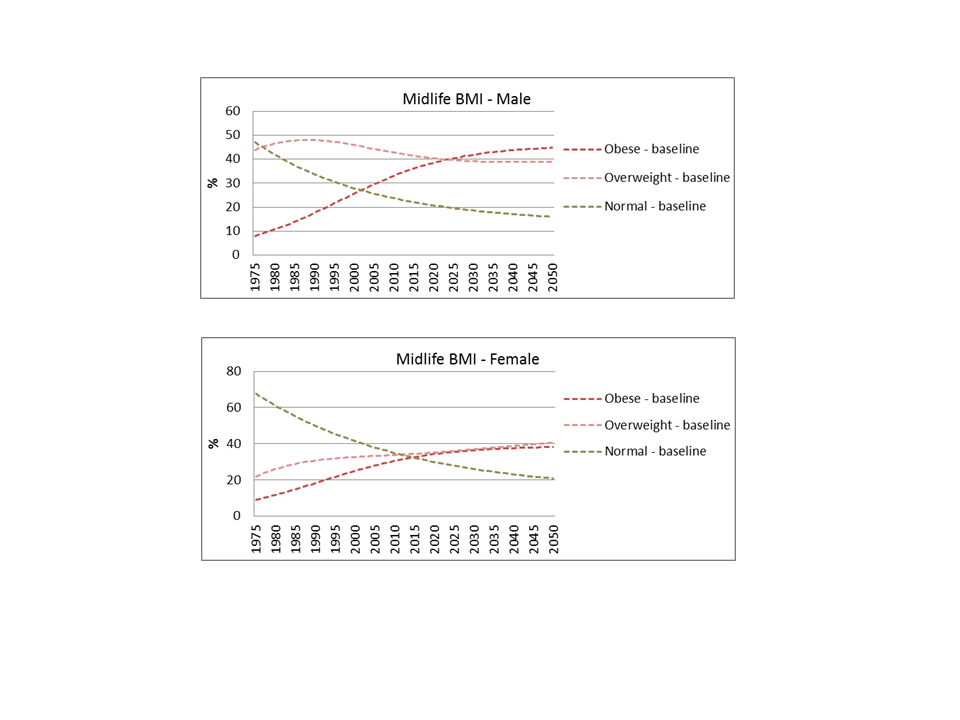

Supplement: Appendix S1 — Baseline trend of prevalence of midlife obesity, overweight and normal weight. (TIF) [file pone.0099305.s001.tif]
